# Acid-base properties of non-protein nitrogen affect nutrients intake, rumen fermentation and antioxidant capacity of fattening Hu sheep

**DOI:** 10.3389/fvets.2024.1381871

**Published:** 2024-03-26

**Authors:** Wenjin Zheng, Hongwei Duan, Liwen Cao, Shengyong Mao, Junshi Shen

**Affiliations:** ^1^Laboratory of Gastrointestinal Microbiology, National Center for International Research on Animal Gut Nutrition, Nanjing Agricultural University, Nanjing, China; ^2^Ruminant Nutrition and Feed Engineering Technology Research Center, College of Animal Science and Technology, Nanjing Agricultural University, Nanjing, China

**Keywords:** urea, ammonium chloride, rumen fermentation, nutrient digestion, antioxidant capacity

## Abstract

This study conducted a comparison of the effects of non-protein nitrogen with different acid-base properties on feed intake, rumen fermentation, nutrient digestion and antioxidant capacity in fattening Hu sheep. Sixteen fattening male sheep (31.43 ± 2.41 kg) with permanent rumen cannulas were randomly assigned to two dietary treatments: 1% urea and 1.78% ammonium chloride (NH_4_Cl, AC). A 42 days experimental period was conducted, with 14 days for adaptation and 28 days for treatment. Daily feed intake was recorded and various samples including feed, feces, rumen fluid, and blood were collected at different time points during the final week. The results indicated that the urea group had significantly higher dry matter intake, average daily gain, and gain efficiency in comparison to the AC group (*p* < 0.01). There was no difference in rumen pH and concentration of ammonia nitrogen between different groups (*p* > 0.05), but the rumen pH of urea group was higher than that of the AC group at 1 and 3 h after feeding (*p* < 0.05). The urea group exhibited higher concentrations of total volatile fatty acids (VFA) and individual VFAs compared to the AC group at all-time points (*p* < 0.01). Compared to the urea group, the intake of all nutrients decreased in the AC group (*p* < 0.01), but the digestibility of dry matter and organic matter increased significantly (*p* < 0.01), and the digestibility of CP had an increasing trend (*p* = 0.06) in the AC group. Additionally, the urea group had lower levels of serum glucagon-like peptide-1, peptide YY, Cl, total protein and globulin than the AC group (*p* < 0.05). The overall levels of HCO_3_^−^, superoxide dismutase, glutathione peroxidase, catalase, albumin/globulin, blood urea nitrogen and total cholesterol in the urea group increased significantly compared to the AC group (*p* < 0.05). It was concluded that adding urea to the high-concentrate diet resulted in increased rumen pH and improved rumen fermentation and growth performance in fattening sheep compared to NH_4_Cl addition. Furthermore, urea addition improved sheep’s antioxidant capacity and maintained their acid-base balance more effectively as compared to NH_4_Cl.

## 1 Introduction

Ruminants play a crucial role in global agricultural production by providing a significant portion of human milk and meat consumption (1). Soybean meal, as the most important protein feed resource for ruminants and other livestock and poultry in China, has faced long-term supply shortages and high import costs (2). Therefore, addressing the pressing need to reduce and replace soybean meal and mitigate feed costs in the face of high-priced protein is imperative for the ruminant industry. The distinctive digestive system of ruminants makes it possible to reduce production costs and dependence on protein resources by adding a minimal quantity of non-protein nitrogen (NPN) to their diet. NPN has been extensively employed in ruminant production in European and American (3, 4), and it exhibits considerable potential for reducing and substituting soybean meal in ruminant feed in China (2).

Depending on their chemical properties, NPNs can be classified into two categories: alkaline NPN, including urea, and diammonium hydrogen phosphate, and acidic NPN, such as NH_4_Cl, ammonium sulfate, ammonium dihydrogen phosphate. Urea, an alkaline NPN, releases free NH_3_ and CO_2_, which can elevate rumen pH (5–7). Studies have shown that appropriate addition of urea in the diet does not negatively affect the DMI, growth performance, nutrient digestion or rumen function of ruminants (8–10). NH_4_Cl, classified as an acidic NPN, generates NH_4_^+^and Cl^−^ ions that have the potential to lower rumen pH (7, 11, 12). Dietary anion-cation difference (DCAD) is commonly used to measure the balance of cations and anions in the diet of animals. Adding NH_4_Cl to the diet can reduce DCAD, modulate diet acidity, and prevent urinary calculus (13, 14). However, excessive NH_4_Cl supplementation can negatively impact animal feed intake and growth performance (15). Consequently, the choice of NPN must be tailored to the diet composition, animal species or physiological stage taking into account the varying acidity and alkalinity of NPN. While previous research has concentrated on the diverse concentrations of NPN, there has been limited exploration into the impact of NPN’s acid-base properties on ruminant growth, rumen fermentation, nutrient utilization, and plasma metabolites.

A stable rumen environment is crucial for the efficient digestion and absorption, as well as for promoting the healthy development of ruminants. In both intensive and extensive production systems, most producers opt to provide their animals with a highly concentrated diet to enhance production performance and attain greater economic benefits (16). Nevertheless, a high-concentrate diet can potentially disturb the rumen fermentation environment, resulting in a decrease in rumen pH and the onset of rumen acidosis after prolonged feeding (17). The disruption of the epithelial barrier and subsequent inflammation, such as gastritis, mastitis, and hoof disease, caused by ruminal acidosis can pose a significant risk to the health of animals (18, 19). Given the current production context and the acid-base properties of NPN, it is hypothesized that the acid-base properties of NPN will initially modify the rumen pH, subsequently influencing the rumen fermentation environment and physiological indicators in blood, ultimately resulting in varied effects on feed intake and growth performance of animals. Therefore, the present study was performed to compare the effects of NPN with different acidity and alkalinity on feed intake, rumen fermentation, and antioxidant capacity in fattening Hu sheep by supplementing their high-concentrate diet with isonitrogenous urea or NH_4_Cl.

## 2 Materials and methods

### 2.1 Animals, diets, and experimental design

Sixteen 5 months fattening male Hu sheep (31.43 ± 2.41 kg) fitted with rumen cannula were assigned into two treatments using a completely randomized design. Two isonitrogenous and isoenergetic diets were prepared using NH_4_Cl and urea, respectively. The diet of the AC group contained 1.78% NH_4_Cl, while the diet of the urea group contained 1% urea. The two diets (Table 1) for fattening sheep were designed to meet their growth requirements (20).

**Table 1:** Ingredients and chemical composition of the experimental diets.

Item	Ammonium chloride	Urea
**Ingredient, % DM**		
Corn silage	20.00	20.00
Peanut vine	20.00	20.00
Corn grain	33.22	34.00
Soybean meal	6.50	6.50
DDGS	11.00	11.00
Wheat bran	5.00	5.00
Urea	0.00	1.00
Ammonium chloride	1.78	0.00
Premix^a^	2.50	2.50
**Nutrient composition, % DM**		
CP	16.48	16.61
NDF	32.44	32.84
ADF	18.89	19.05
EE	4.07	4.13
Ash	7.78	7.80
DE, MJ/kg	13.43	13.54
Ca	0.89	0.89
P	0.48	0.44
Mg	0.36	0.38
K	1.12	1.14
S	0.14	0.16
Na	0.42	0.37
Cl	1.61	0.67
DCAD^b^, mEq/kg	−72.74	164.35

a Formulated to provide (per kilogram of premix): 300 g of salt, 150 g of Ca, 30 g of P, 250,000 IU of vitamin A, 80,000 IU of vitamin D3, 2,400 IU of VE, 1,000 mg of Cu, 1,500 mg of Fe, 1,200 mg of Mn, 2,000 mg of Zn, 30 mg of I, 10 mg of Se, 12 mg of Co.

b Dietary cation-anion difference calculated using the equation: [(mEq of Na^+^+ mEq of K^+^) − (mEq of Cl^−^ + mEq of S^2−^)].

This study spanned a period of 42 days, comprising of a 14 days adaptation phase and a subsequent 28 days dietary treatment phase. Throughout the duration of the experiment, all sheep were individually housed and fed twice a day at 07:30 h and 17:30 h respectively, with free access to drinking water. The quantity of diet dispensed was tailored to their daily feed intake, with a surplus of 5–10% being ensured.

### 2.2 Sampling and measurement

Weights of each sheep were measured before morning feeding for three consecutive days before the trial, and subsequently on days 20, 21, and 22, in order to determine the average daily gain (ADG). The diet offered and orts were recorded daily to measure the average daily dry matter intake (DMI). ADG/DMI was used to calculate sheep gain efficiency.

Dietary samples (500 g) were collected at the initial, intermediate, and final stages of the trial, respectively. Fecal samples were collected from each sheep at 08:00 h and 18:00 h on days 22, 23, and 24. The feed and fecal samples of per sheep were subsampled and then stored at −20°C until analysis. All samples were thawed and dried for 48 h at 65°C at the end of the experiment. A Cyclotec mill (Tecator 1093; Tecator AB, Höganäs, Sweden) was used to grind the dried samples through a 1 mm screen before analysis. All samples were analyzed for dry matter (DM), organic matter (OM), crude protein (CP) and ether extract (EE) using the standard procedure of AOAC (21). According to Van Soest et al. (22), contents of acid detergent fiber (ADF) and neutral detergent fiber (NDF) were measured. The acid-insoluble ash (AIA) in the diet and fecal samples was measured and used as an internal marker to determine the apparent total tract nutrient digestibility (23). The dietary contents of Ca, P, Mg, S and Na were analyzed using Inductively Coupled Plasma-Optical Emission Spectrometer (Thermo Scientific, 7400), while the dietary contents of K and Cl were measured by commercial kits (Jiancheng Bioengineering Institute, Nanjing, China). The DCAD were calculated using the formula: mEq/kg of DM = (mEq of Na^+^ + mEq of K^+^) − (mEq of Cl^−^ + mEq of S^2−^) (24).

Rumen fluid was collected through the rumen cannula at 0 h, 1 h, 3 h and 6 h after morning feeding on day 28, and was mixed and filtered through four layers of gauze. The values of rumen pH were instantly measured using a portable pH meter (pHS-10). One mL of each ruminal fluid sample was stored at −20°C and subsequently used to analyze the ammonia concentration by a colorimetric method (25). Another 1 mL of each ruminal fluid sample was supplemented with 0.2 mL of 25% HPO_3_, which was preserved to analyze VFA concentrations by gas chromatography (7890A, Agilent, Santa Clara, CA, United States) as described by Mao et al. (26).

Blood samples (9 mL) were collected from sheep’s jugular vein using heparinized vacutainer tubes at 0 h, 1 h, 3 h and 6 h after morning feeding on day 26. The samples were centrifuged at a speed of 3,000 × g for 15 min in order to obtain the plasma, which was then stored at −20°C for later analysis. The concentrations of blood urea nitrogen (BUN), glucose, total protein (TP), albumin, globulin, total cholesterol (TCHO), triglyceride (TG), and superoxide dismutase (SOD) were determined using an automatic biochemistry analyzer (Beckman Coulter AU680, United States). The hormone indexes, namely insulin, glucagon-like peptide-1 (GLP-1), and peptide YY (PYY), were analyzed using the ELISA kits (Jiangsu Meimian Industrial Co., Ltd., Jiangsu, China). Glutathione peroxidase (GSH-Px), catalase (CAT) and malondialdehyde (MDA) were measured by commercial kits (Jiancheng Bioengineering Institute, Nanjing, China).

### 2.3 Statistical analyses

SAS 9.4 (SAS Institute Inc., Cary, NC, United States) were performed for data analysis using the MIXED procedure. Preliminary data examination revealed that all dependent variables were normally distributed. When data for growth performance (DMI, BW, ADG, gain efficiency), nutrient digestion were analyzed, a fixed effect of treatment and a random effect of sheep were included in the statistical model. Data for rumen fermentation parameters and blood indicators were analyzed with time as repeated measures using covariance type of autoregressive (1), which provides the best fit according to Akaike’s information criterion. The statistical model incorporated treatment, time, and the interaction of treatment × time as a fixed effect, while sheep were considered a random effect. A statistically significant difference was considered to exist when the *p*-values were <0.05, and trends were declared at 0.05 ≤ *p* < 0.10. Unless otherwise stated, all values are least-squares means.

## 3 Results

### 3.1 Dietary chemical composition, dry matter intake and growth performance

**Figure 1:**
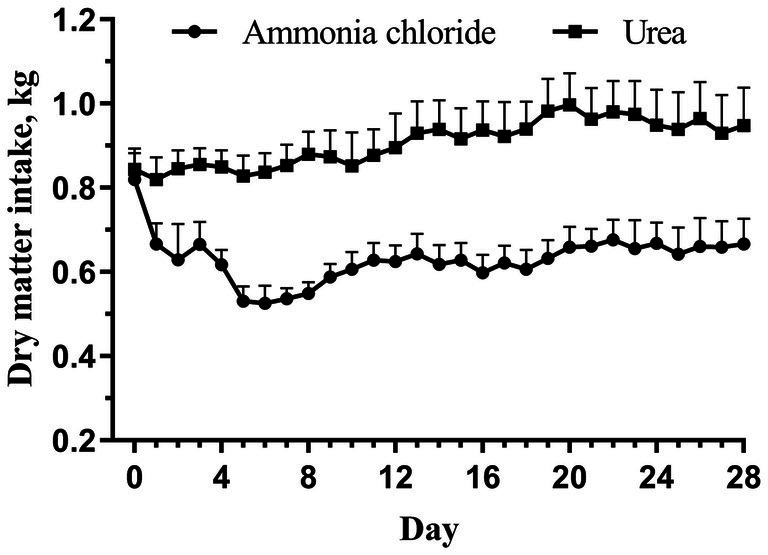
Dynamic changes of feed intake in fattening sheep fed with ammonium chloride and urea. Values are least squares means ± SE (*n* = 8).

The chemical composition of the AC group’s diet was comparable to that of the urea group, with the exception of Cl ion levels (Table 1). Consequently, the calculated DCADs for the AC and urea groups were −72.74 and 164.35, respectively. As shown in Figure 1, no significant difference was observed in initial DMI between the AC and urea groups (*p* > 0.05). Nevertheless, the DMI of the AC group decreased significantly from day 1 to day 28 when compared to the urea group (*p* < 0.05). Additionally, there was no significant difference in the initial BW of sheep between different treatments (Table 2; *p* = 0.90). However, the final BW, ADG, DMI and G: F of the urea group were significantly higher compared to the AC group (*p* < 0.05).

**Table 2:** Comparative effects of ammonium chloride and urea supplementation on growth performance in fattening sheep.

Item	Ammonium chloride	Urea	SEM	*p*-value
**BW, kg**				
Initial	31.39	31.55	0.975	0.90
Final	32.63	35.75	0.959	0.04
ADG, g/day	58.9	200.0	17.23	<0.01
DMI, g/day	651.2	898.7	56.18	<0.01
G: F	0.089	0.222	0.018	<0.01

### 3.2 Rumen fermentation characteristics

**Figure 2:**
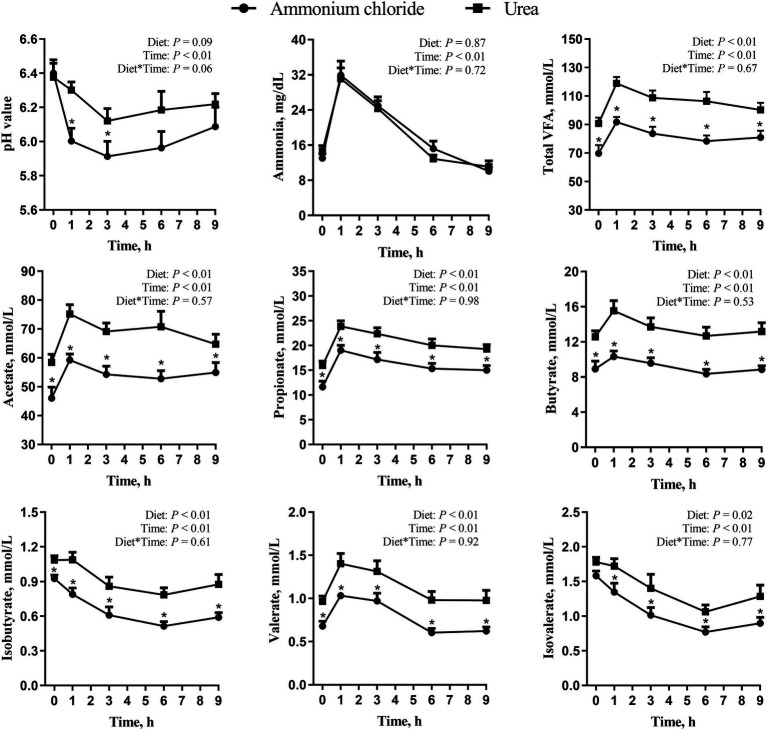
Comparative effects of ammonium chloride and urea supplementation on rumen fermentation parameters in fattening sheep. Values are least squares means ± SE (*n* = 8).

As shown in Figure 2, there were no significant interaction effects of treatment and time on rumen pH, ammonia concentrations, and VFA (*p* > 0.05). Rumen pH in the urea group was higher at 1 and 3 h after morning feeding compared to the AC group (*p* < 0.01). Furthermore, time had a significant impact on rumen pH, with both groups experiencing the lowest levels at 3 h after feeding (*p* < 0.01). There was no difference in the concentration of ammonia nitrogen between different treatments at all-time points (*p* > 0.05). However, both concentrations of ammonia nitrogen in the two groups exhibited a rising trend initially, followed by a decline, with the maximum value being attained at 1 h after morning feeding (*p* < 0.01). The levels of individual and total VFA in the urea group were higher than those in the AC group at all-time points (*p* < 0.05).

### 3.3 Nutrients intake and apparent digestibility

Sheep in the AC group consumed significantly less DM, OM, CP, NDF, ADF, and EE in comparison to the urea group (*p* < 0.01; Table 3). However, the apparent digestibility of DM and OM increased (*p* < 0.01) and that of CP tended to increase (*p* = 0.06) in the AC group. No significant difference was shown in the apparent digestibility of NDF, ADF, and EE between different treatments (*p* > 0.05).

**Table 3:** Comparative effects of ammonium chloride and urea supplementation on nutrients intake and apparent total tract digestibility of nutrients in fattening sheep.

Item	Ammonium chloride	Urea	SEM	*p*-value
**Intake, g/day**				
DM	655.7	976.4	73.61	<0.01
OM	604.7	900.2	67.87	<0.01
CP	108.1	162.2	12.19	<0.01
NDF	212.7	320.6	24.07	<0.01
ADF	123.9	186.0	13.98	<0.01
EE	26.7	40.3	3.02	<0.01
**Digestibility, %**				
DM	73.6	71.5	0.48	<0.01
OM	75.9	73.6	0.50	<0.01
CP	75.8	74.5	0.42	0.06
NDF	53.9	54.8	1.30	0.62
ADF	51.7	54.5	1.42	0.19
EE	90.7	90.4	0.34	0.52

### 3.4 Hormone, antioxidant index and plasma metabolites

**Figure 3:**
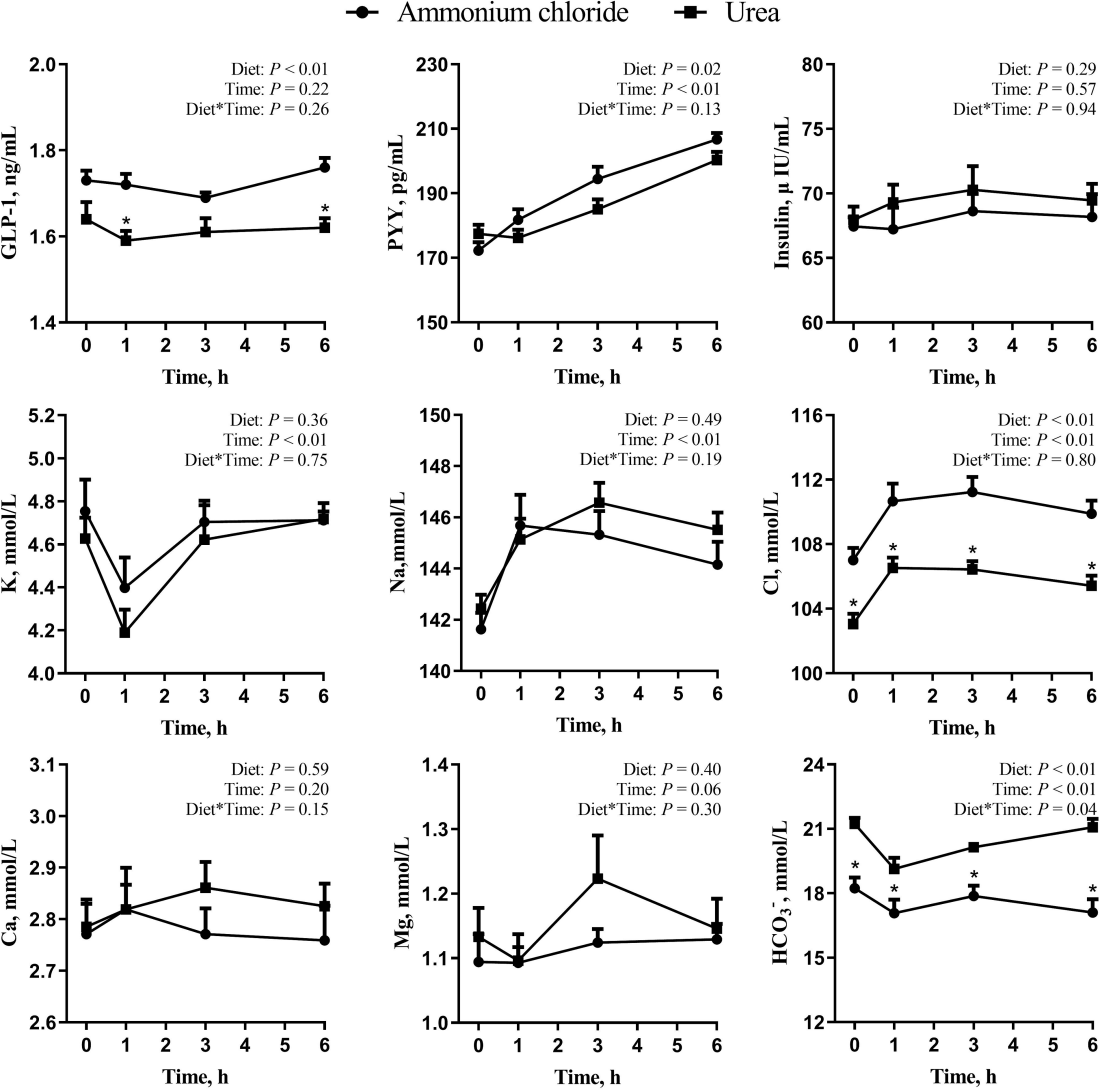
Comparative effects of ammonium chloride and urea supplementation on blood hormones and ions in fattening sheep. Values are least squares means ± SE (*n* = 8).

As shown in Figure 3, there was a significant treatment × time interaction effect on HCO_3_^−^ (*p* < 0.05), while no interaction effects were observed on GLP-1, PYY, insulin, K, Na, Cl, Ca, and Mg (*p* > 0.05). Additionally, the overall levels of GLP-1 and PYY in the AC group increased significantly (*p* < 0.05), and the level of Cl in the AC group was higher at all-time points (*p* < 0.01) in comparison to the urea group. In contrast, the level of HCO_3_^−^ in the AC group decreased significantly at all-time points in comparison to the urea group (*p* < 0.01). No difference was observed in insulin, K, Na, Ca and Mg between the two groups (*p* > 0.05).

**Figure 4:**
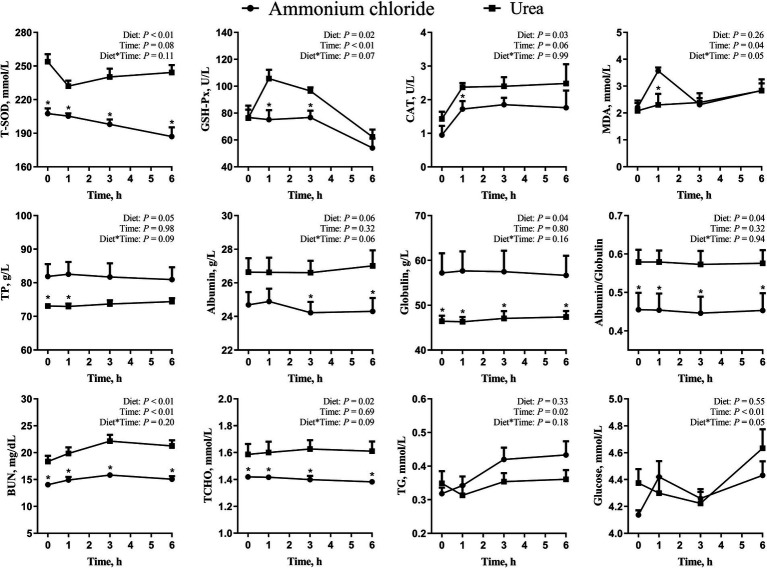
Comparative effects of ammonium chloride and urea supplementation on antioxidant indices and blood parameters in fattening sheep. Values are least squares means ± SE (*n* = 8).

As shown in Figure 4, there were significant treatment × time interaction effects on MDA and glucose levels (*p* < 0.05), while no interaction effects on T-SOD, GSH-Px, CAT, TP, albumin, globulin, albumin/globulin, BUN, TCHO, and TG (*p* > 0.05). Compared with the AC group, the overall levels of TP and globulin decreased significantly (*p* < 0.05), while the overall levels of SOD, GSH-Px, CAT, albumin, albumin/globulin, BUN and TCHO increased significantly in the urea group (*p* < 0.05). Additionally, the concentration of MDA in the urea group decreased significantly at 1 h (*p* < 0.05) compared to the AC group. No significant difference was observed in glucose and TG between different treatments (*p* > 0.05).

## 4 Discussion

### 4.1 Dry matter intake and growth performance

Addition dosage of NPN affects the DMI of ruminants (4, 27). Supplementation with 1% urea has been proved by previous studies to improve the DMI of ruminants without negative effects on growth performance (9, 28). In contrast, several studies have demonstrated that high levels of NH_4_Cl, whether added to the diet of cattle (85.1 g/day equating to approximately 0.89% DM) or injected into the rumen of dairy cows (300 g/day equating to approximately 1.76% DM), can reduce DMI and growth performance of ruminants (15, 29). Therefore, it is reasonable to speculate that the addition of 1.78% NH_4_Cl to satisfy the isonitrogenous conditions equivalent to 1% urea in this study exceeded the appropriate range, resulting in a notable reduction in the DMI and growth performance of sheep.

NH_4_Cl can dissociate into NH_4_^+^ and Cl^−^, and dietary addition of NH_4_Cl could reduce DCAD (30). A low or negative DCAD can result in a decrease in rumen pH and HCO_3_^−^ levels in the blood (31), which was in line with the present study. A reduction in DCAD has been associated with a decrease in ruminant DMI according to several studies (32, 33). Additionally, Zimpel et al. (34) have reported that ruminants fed with a low DCAD would experience metabolic acidosis, which may result in the observed decrease in DMI. In this study, the rumen pH of sheep in the AC group experienced a decline below 6.0 for over 5 h from 1 h after morning feeding, accompanied by a decrease in the level of HCO_3_^−^ in the blood. This occurrence may suggest that the sheep in the AC group were in a state of metabolic acidosis, leading to a serious decline in their DMI. Furthermore, it was observed in this study that sheep in the AC group exhibited heightened levels of hormones such as GLP-1 and PYY in their serum, which can inhibit their feed intake (35, 36). This discovery has contributed supplementary knowledge regarding the reduction in ruminant feed consumption.

### 4.2 Nutrients intake and apparent digestibility

The growth performance of animals is directly influenced by the intake and apparent digestibility of nutrients (37). According to the present study, sheep belonging to the AC group exhibited reduced feed consumption, consequently leading to insufficient intake of essential nutrients. Nevertheless, the AC group demonstrated an enhanced ability to digest DM, OM, and CP, suggesting the animals’ efforts to enhance nutrient digestion and absorption in order to meet their metabolic requirements. However, despite the observed improvement in digestion, it was not enough to compensate for the inadequate nutrient intake, leading to inferior growth performance in comparison to the urea group. In addition, the elevated feed intake observed in the urea group may improve the rate of diet flow through the gastrointestinal tract, leading to insufficient interaction between the diet and digestive enzymes, ultimately causing incomplete digestion and absorption of a portion of the diet. According to Castaneda et al. (38), the intake of DM, OM, CP, NDF, ADF and EE significantly decreased, while the digestibility of DM, OM, CP and NDF significantly increased with the progressive substitution of urea with isonitrogenous NH_4_Cl in the diet of Holstein bulls, which showed the same trend as the results of this study.

### 4.3 Rumen fermentation characteristics

The rumen is a vital organ for ruminants to digest and absorb nutrients. The optimal internal environment of the rumen, including pH and ammonia nitrogen concentration, plays an essential role in the absorption of ammonia through the rumen epithelium and the proliferation of rumen microorganisms (39). Shen et al. (7) found in an *in vitro* fermentation trial that urea addition increased rumen pH, while NH_4_Cl addition slightly reduced rumen pH, which aligns with the findings of this study. It is noteworthy that high-concentrate diets have been extensively utilized in contemporary intensive production to enhance growth performance and economic efficiency. However, this may lead to a decline in the rumen pH and an augmented likelihood of rumen acidosis, which is detrimental to the healthy development of ruminants. In a previous study, Xu et al. (9) found that rumen pH increased with the increase of urea addition to a high-concentrate diet, indicating that urea addition can mitigate the low rumen pH caused by high-concentrate diets and diminish the risk of rumen acidosis.

Ammonia concentration in *in vivo* rumen fluids is predominantly influenced by the interplay among the rate of ammonia production, microbial utilization, and absorption through the rumen epithelium. The current study revealed that the AC group, which exhibited a lower CP intake but comparable digestibility to the urea group, suggested a decrease in ammonia production. Nevertheless, the AC group exhibited a comparable ammonia nitrogen concentration to the urea group, potentially due to a reduction in ammonia absorption through the rumen epithelium and a decrease in microbial utilization for the production of microbial protein. It has been proved that pH is a crucial determinant of ammonia absorption of rumen epithelium (40). The rate of ammonia absorption through the rumen epithelium increases with the increase in rumen pH (41). Therefore, in the present study, the decrease in rumen pH of the AC group indicated a reduced absorption of ammonia through the rumen epithelium. Besides, a low rumen pH is known to be unfavorable for rumen microbial fermentation (42), which may prevent microorganisms from synthesizing microbial protein from ammonia. The reduced ammonia utilization by microbes in the AC group is substantiated by the decreased pH and total VFA concentration.

### 4.4 Hormone, antioxidant index and plasma metabolites

Both GLP-1 and PYY serve as satiety signals that can reduce animal feed intake by modulating intestinal movement and increasing bodily satiety (43, 44). It was found by Relling et al. (45) that the serum GLP-1 level of animals increased and their feed intake decreased significantly by injecting GLP-1 into sheep, indicating that blood hormones related to feed intake play a crucial role in regulating sheep’s DMI. Therefore, the substantial reduction in DMI observed in the AC group may be attributed to the increase in GLP-1, PYY, and other hormones in the blood. According to Ahmad and Sarwar (46), a positive correlation was observed between dietary Cl^−^ levels and blood Cl^−^ levels in broilers, and high levels of Cl^−^ were found to reduce the level of HCO_3_^−^ in the blood, which is consistent with the present findings in sheep. Furthermore, HCO_3_^−^ can not only improve the buffering capacity and keep the acid-base balance of the blood, but also serve as an indicator of metabolic acidosis in animals (47). In the present study, sheep in the AC group exhibited reduced serum HCO_3_^−^ levels, indicating a potential decrease in blood buffering capacity and the possibility of metabolic acidosis, which may have contributed to a reduced appetite.

BUN mainly comes from the absorption of ammonia nitrogen through the rumen epithelium and the decomposition of tissue protein, with a strong relationship to the concentration of ammonia nitrogen in the rumen (48). While ammonia nitrogen levels in the rumen were comparable between different groups in this study, a variation in rumen pH was observed. A study conducted by Abdoun et al. (40) revealed that an increase in rumen pH led to a corresponding rise in the overall quantity of ammonia absorbed through the rumen epithelium. Consequently, the urea group exhibited a greater rate of absorption of ammonia through the rumen epithelium than the AC group, leading to a significantly higher BUN level in the former. These findings align with those of Castaneda et al. (38), who found a linear decrease in BUN levels with an increase in isonitrogenous NH_4_Cl substitution for urea in the diet of Holstein bulls.

Both T-SOD and GSH-Px are endogenous antioxidant enzymes that safeguard the body against oxidative stress and injury (49). In contrast, MDA is the final product of lipid peroxidation, and it can give an indication of cell damage (50). In addition, the levels of albumin and globulin can serve as markers of the immune status of the body (51). It has been shown that the levels of T-SOD, CAT, and GSH-Px in serum decrease, while the level of MDA increases when sheep are under stress (52). In this study, sheep in the AC group exhibited reduced levels of T-SOD, CAT, and GSH-Px, and an increased level of MDA, indicating a decline in their antioxidant capacity and the potential occurrence of oxidative stress following the consumption of a diet containing NH_4_Cl. Under such conditions, the oxidation of albumin may result in a loss of its activity (53). Concurrently, in an effort to mitigate oxidative harm, sheep in the AC group may elevate globulin levels and enhance their own immunity, potentially leading to hyper humoral immunity. In a previous study, De and Dey found that goats with lower antioxidant capacity displayed a decreased level of albumin, while experiencing an elevation in globulin level (54), which is consistent with the present study.

## 5 Conclusion

The addition of isonitrogenous 1% urea and 1.78% NH_4_Cl resulted in variations in feed intake, rumen fermentation, nutrient digestion and antioxidant capacity of fattening sheep. Adding urea to the basal high-concentrate diet led to an increased rumen pH and an enhancement of rumen fermentation and growth performance in fattening sheep, as compared to NH_4_Cl addition. Urea addition was found to be more conducive to maintaining the acid-base balance and enhancing the antioxidant capacity of sheep in comparison to NH_4_Cl.

## Data availability statement

The original contributions presented in the study are included in the article/supplementary material, further inquiries can be directed to the corresponding author.

## Ethics statement

All animal protocols were approved by the Animal Care and Use Committee of Nanjing Agricultural University (Protocol number: SYXK2017-0007). The study was conducted in accordance with the local legislation and institutional requirements.

## Author contributions

WZ: Data curation, Formal analysis, Investigation, Visualization, Writing – original draft. HD: Investigation, Visualization, Writing – original draft. LC: Investigation, Visualization, Writing – original draft. SM: Methodology, Resources, Supervision, Writing – review & editing. JS: Conceptualization, Data curation, Funding acquisition, Methodology, Project administration, Resources, Writing – review & editing.
